# Sex and BMI as predictors of pill residue in dysphagia: a multivariate analysis

**DOI:** 10.1038/s41598-026-43307-z

**Published:** 2026-03-08

**Authors:** Ayako Nakane, Mariko Ando, Yu Yoshizumi, Shinya Mikushi, Kazuo Motomura, Haruka Tohara

**Affiliations:** 1 Dentistry and Oral Surgery, Community Healthcare Organization, Tokyo Shinjuku Medical Center, 5-1, Tsukudo-cho, Shinjuku-ku, 162-8543 Tokyo Japan; 2 Department of Dysphagia Rehabilitation, Division of Gerontology and Gerodontology, Graduate School of Medical and Dental Sciences, Institute of Science Tokyo, 1-5-45, Yushima, Bunkyo-ku, 113-8549 Tokyo Japan; 3 Oral Surgery, Saitama Red Cross Hospital, Japanese Red Cross Society, 1-5, Shintoshin, Chuo-ku, 330-8553 Saitama Japan; 4 Morimoto Dental Clinic, 5-11-66 Nameshi, Nagasaki city, 852-8061 Nagasaki Japan; 5 Department of Gerodontology and Oral Rehabilitation, Division of Gerontology and Gerodontology, Graduate School of Medical and Dental Sciences, Institute of Science Tokyo, 1-5-45, Yushima, Bunkyo-ku, 113-8549 Tokyo Japan

**Keywords:** Body mass index, Piriform sinus, Deglutition disorders, Pill residue, Retrospective studies., Health care, Medical research

## Abstract

We aimed to explore the specific challenges encountered by individuals with dysphagia when taking oral medications, focusing on the types of dysphagia that impede pill swallowing and the relationship between body mass index (BMI) and pill residue. We retrospectively reviewed 70 patients who underwent videofluoroscopic swallowing studies at the Department of Dysphagia Rehabilitation, Tokyo Medical and Dental University Dental Hospital between May 2013 and March 2020. Patients were assessed for pill residue in various anatomical locations, including the mouth, epiglottic vallecula, and piriform sinus. Patient demographics, BMI, functional oral intake scale scores, and clinical histories were collected. Pill residue was most commonly observed in the epiglottic vallecula (17% of patients), followed by the piriform sinus (10%) and mouth (9%). Males were more likely to have tablet residue in the epiglottic vallecula (*p* = 0.047), and a lower BMI was associated with increased pill residue in the piriform sinus (*p* = 0.025). Multivariate analysis identified sex as a significant predictor of epiglottic pill residue (*p* = 0.031), whereas a lower BMI was associated with pill residue in the piriform sinus (*p* = 0.016). Sex and BMI significantly influenced pill residue in patients with dysphagia; males and individuals with a lower BMI (< 18.5 kg/m^2^) were at higher risk.

## Introduction

Dysphagia, or difficulty swallowing, is a significant clinical concern, particularly among aging populations and individuals with neurological disorders^1–3^. It can severely impact a person’s ability to safely consume food and liquids, often necessitating dietary modifications such as altering meal consistency or adding thickening agents to liquids to prevent aspiration^4^. While these dietary adjustments are commonly addressed, challenges associated with swallowing oral medications are often overlooked despite their critical role in managing chronic conditions and ensuring patient adherence with treatment regimens^5,6^.

The gold standard for diagnosing dysphagia is the videofluoroscopic swallowing study (VFSS), a radiographic examination that allows for the visualization of the entire swallowing process from the mouth to the stomach^7^. VFSS is particularly valuable for tracking the movement of barium-prepared simulated pills and providing continuous feedback on swallowing mechanics^8^.

Despite the well-recognized impact of dysphagia on food and liquid intake, specific challenges related to swallowing oral medications have not been sufficiently addressed in clinical practice. Notably, some studies have reported associations between low body mass index (BMI) and dysphagia; however, significant direct correlations between BMI and specific swallowing measures have yet to be explored^9^. Patients with dysphagia who experience difficulty taking oral medication are at risk of noncompliance, which can exacerbate their underlying conditions and result in poorer health outcomes. Clinical swallowing disorders or dysphagia manifest differently in men and women, but the evidence remains sparse and inconclusive^10,11^. Given the importance of safe and effective medication administration, identifying dysphagia types that contribute to pill-swallowing difficulties and developing targeted interventions based on patients’ BMI and sex are critical.

This study aimed to explore the specific challenges faced by individuals with dysphagia when taking oral medications, focusing on the types of dysphagia that interfere with pill swallowing. We assessed patients’ swallowing mechanics and investigated the relationship between pill-swallowing ability and BMI as well as sex using VFSS.

## Results

### Patient demographics

Patients’ demographics and clinical characteristics are summarized in Table 1. The study included 70 patients, with a slight male predominance (57% male, 43% female). The median age of the participants was 77.0 years (interquartile range [IQR] 70.3–83.0) years, with no significant age difference between males 78.5 years (IQR 68.8–81.3) and females 75.5 (IQR 71.0–84.0) years (*p* = 0.370). The median BMI was 20.0 kg/m^2^ (IQR 17.9–22.9), with females having a slightly higher BMI 21.0 (IQR 17.8–23.2) than males 19.9 (IQR 18.1–21.9), though this difference was not statistically significant (*p* = 0.221). Notably, 17% of the participants had a BMI of less than 18.5, indicating undernutrition, with a higher proportion of females (10%) than males (7%), although this difference was not statistically significant (*p* = 0.089) (Table 1). Regarding medical history, cerebrovascular disease (21%) and Parkinson’s disease (15%) were the most common underlying conditions. Dementia was more prevalent in females (7%) compared with males (3%), though the overall distribution of illnesses between sexes did not show significant differences (*p* = 0.255).

**Table 1:** Characteristics of the subjects.

	All *N* = 70	Male *N* = 40(57%)	Female *N* = 30(43%)	*P*-value
Age (median IQR) (y)	77.0 (70.3–83.0)	78.5 (68.8–81.3)	75.5(71.0–84.3)	0.370
BMI (*N* = 59) (median IQR) (kg/m^2^)	20 0.0(17.9–22.9)	19.9 (18.1–21.9)	21.0(17.8–23.2)	0.221
BMI<18.5 N(%)	12(17)	5(7)	7(10)	0.089
Medical history N(%)				0.255
Cerebrovascular disease	15(21)	9(13)	6(9)	
Parkinson’s disease	11(15)	5(7)	6(8)	
Dementia	7(10)	2(3)	5(7)	
Gastrointestinal disorders	7(10)	5(7)	2(3)	
Neurological diseases	5(7)	4(6)	1(1)	
Psychiatric disorders	4(6)	1(1)	3(4)	
Pneumonia	4(6)	1(1)	3(4)	
Respiratory diseases	3(4)	3(4)	0(0)	
Spinal canal stenosis	3(4)	3(4)	0(0)	
Others	11(15)	7(10)	4(6)	
FOIS at the time of the first medical examination N(%)				0.093
FOIS7	42(60)	26(37)	16(23)	
FOIS6	11(16)	7(10)	4(6)	
FOIS5	9(13)	4(6)	5(7)	
FOIS4	6(9)	3(4)	3(4)	
FOIS2	2(3)	0(0)	2(3)	
Primary complaint at the time of the first medical examination N(%)				0.004
Cough while eating	35(50)	25(36)	10(14)	
Weight loss	3(4)	1(1)	2(3)	
Difficulty swallowing	13(19)	8(11)	5(7)	
Catching in the throat (chest)	13(19)	5(7)	8(11)	
Phlegm in the mouth	3(4)	1(1)	2(3)	
Want to eat by mouth	3(4)	0(0)	3(4)	

The FOIS scores at the first examination indicated that 60% of patients were classified at the highest level (FOIS 7), with males having higher FOIS scores (37%) than females (23%). The distribution of FOIS scores did not show a statistically significant difference between sexes (*p* = 0.093).

The primary complaint at the first examination showed a significant sex difference (*p* = 0.004). The most common complaint was cough while eating (50%), with a higher prevalence in males (36%) than females (14%). Other complaints included difficulty swallowing (19%), catching in the throat or chest (19%), and weight loss (4%). Of the total cohort, only 4% underwent percutaneous endoscopic gastrostomy; all were female and desired oral intake.

### Presence and location of residual pills

**Fig. 1:**
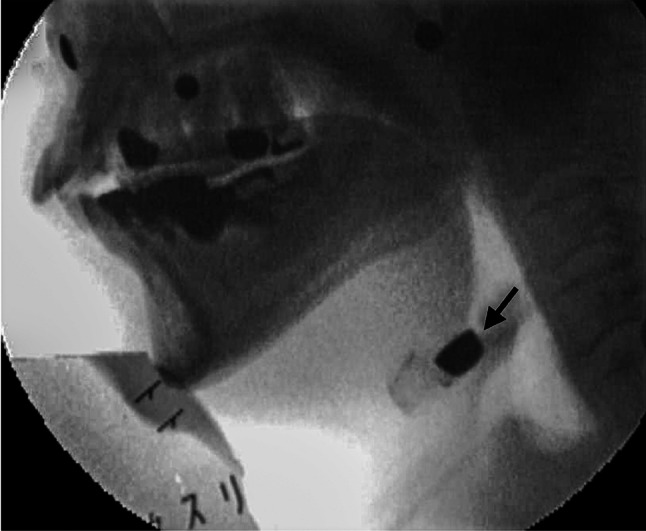
Radiograph, lateral view, showing pill residue in the epiglottis. The arrow indicates the pill residue.

**Fig. 2:**
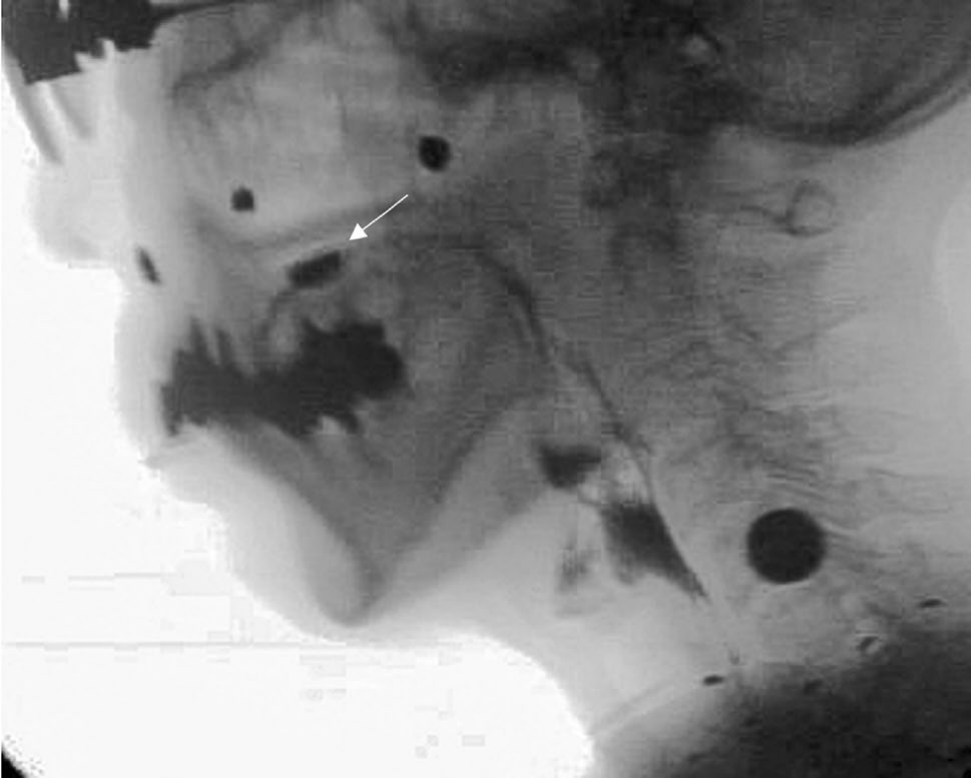
Radiograph, lateral view, showing pill residue in the mouth. The arrow indicates the pill residue.

**Fig. 3:**
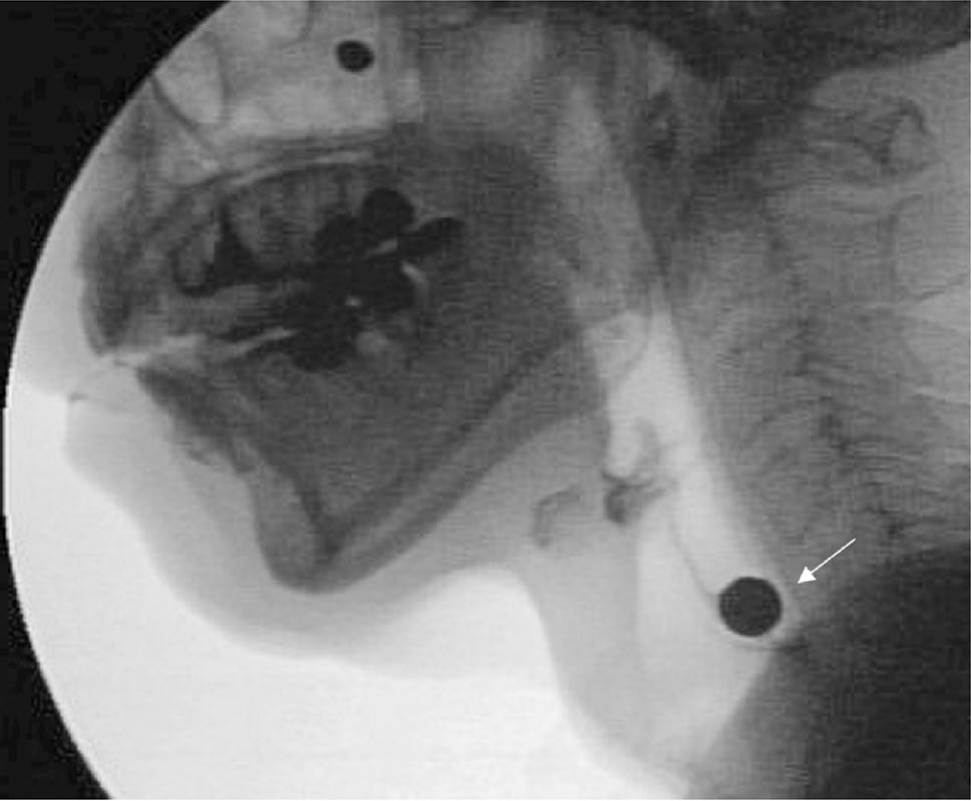
Radiograph, lateral view, showing pill residue in the piriform sinus. The arrow indicates the pill residue.

The results of the VFSS are summarized in Table 2, highlighting the presence or absence of residual simulated pills at various anatomical locations. No one accidentally aspirated the barium placebo pills, but 19% experienced aspiration of the barium solution during administration. Pill residues were most commonly observed in the epiglottis (Fig. 1), 17% (10 males and 2 females) showing pill residue, whereas 83% (30 males and 27 females) showed no pill residue in this area. In the mouth, pill residue was observed in 9% (3 males and 3 females) (Fig. 2), while 91% (37 males and 27 females) had no pill residue. Additionally, 10% (5 males and 2 females) exhibited pill residue in the piriform sinus (Fig. 3), whereas 90% (35 males and 27 females) showed none.

**Table 2:** Result of VFSS analysis (N=70).

		Pill residue (M・F)	No residue (M・F)
Location of pill residue	Mouth%(N)	9(3・3)	91(37・27)
	Epiglottic vallecula %(N)	17(10・2)	83(30・27)
	piriform sinus %(N)	10(5・2)	90(35・27)

		Aspiration(M・F)	No aspiration(M・F)
Presence or absence of aspiration	Pill % (N)	0	100(40・29)*
	Barium liquid % (N)	19(8・5)	81(32・24)

*One participant was completely unable to swallow the pill, so no further tests were conducted, resulting in N=69.

### Factors influencing residuals

Table 3 presents a comparative analysis of demographic and clinical characteristics between patients with pill residue (*n* = 24) and those without (*n* = 46), as observed on VFSS. Patients in the pill residue group were older, with a median age of 81.0 (IQR 73.5–84.0) years compared to 74.5 (IQR 68.3–81.0) years in the non-residue group, but the difference was non-significant (*p* = 0.052). There was a higher proportion of males in the pill residue group (71%) than in the non-residue group (50%), though this difference was not statistically significant (*p* = 0.077).

**Table 3:** Comparison between groups with and without pill residues.

	Residue (*N*=24)	No residue (*N*=46)	*P* value
Age (median IQR) (y)	81.0(73.5–84.0)	74.5(68.3–81.0)	0.052
Sex M(%)	17(71)	23(50)	0.077
BMI (median IQR) (kg/m^2^)	19.0(17.5–22.8)	20.1(18.1–23.0)	0.376
BMI < 18.5 N(%)	22(27)	37(16)	0.244
Medical history N(%)	24(34)	46(66)	0.053
Cerebrovascular disease	8(11)	7(10)	
Parkinson’s disease	4(6)	7(10)	
Dementia	2(3)	5(7)	
Gastrointestinal disorders	3(4)	4(6)	
Neurological diseases	1(1)	4(6)	
Psychiatric disorders	0(0)	4(6)	
Pneumonia	3(4)	1(1)	
Respiratory diseases	2(3)	1(1)	
Spinal canal stenosis	0(0)	3(4)	
Others	1(1)	10(14)	
FOIS at the time of the first medical examination N(%)			0.019
FOIS7	12(17)	30(43)	
FOIS6	3(4)	8(11)	
FOIS5	3(4)	6(9)	
FOIS4	4(6)	2(3)	
FOIS2	2(3)	0(0)	
Primary complaint at the time of the first medical examination N (%)			0.945
Cough while eating	13(19)	22(31)	
Weight loss	1(1)	2(3)	
Difficulty swallowing	4(6)	9(13)	
Catching in the throat (chest)	2(3)	11(16)	
Phlegm in the mouth	2(3)	1(1)	
Want to eat by mouth	2(3)	1(1)	

The median BMI was slightly lower in the pill residue group at 19.0 kg/m² (IQR: 17.5–22.8) compared to 20.1 kg/m² (interquartile range: 18.1–23.0) in the non-residue group; however, this difference was not statistically significant (*p* = 0.376). Similarly, the percentage of patients with a BMI < 18.5 kg/m² was not significantly different between the groups (27% vs. 16%, *p* = 0.244).

A significant difference was not observed in the medical history, with 34% of the pill residue group having a medical history compared with 66% of the non-residue group (*p* = 0.053). Specific conditions such as cerebrovascular disease, Parkinson’s disease, and dementia were present in both groups; however, these differences were not statistically significant.

Patients in the pill residue group had significantly lower FOIS scores at the first examination, indicating a lower level of oral intake (*p* = 0.019). Specifically, 17% of the pill residue group had a FOIS score of 7 (full oral intake) compared to 43% in the non-residue group.

No significant differences were observed in the primary complaints at the first examination between the two groups. Complaints such as coughing while eating, difficulty swallowing, and throat catching were similarly distributed, with no statistically significant differences.

Table 4 presents a comparison between the groups with and without pill residue in three anatomical locations: the mouth, epiglottic vallecula, and piriform sinus. For pill residues in the mouth, 9% (*n* = 6) of patients had pill residue, whereas 91% (*n* = 64) did not. The median age of patients with mouth residues was slightly higher 80.0(IQR 71.0–83.0)years compared to those without pill residues 76.0(IQR 70.0–83.3.0.3) years, though this was not statistically significant (*p* = 0.417). There was no significant difference in the sex distribution between the groups (*p* = 0.517).

**Table 4:** Comparison between groups of analogue pill residues.

	Location of residue								
	Mouth			Epiglottic vallecula			Piriform sinus		
	Residue *N* = 6(9%)	No residue *N* = 64(91%)	*P*	Residue *N* = 12(17%)	No residue *N* = 57(83%)	*P*	Residue *N* = 7(10%)	No residue *N* = 62(90%)	*P*
Age (median IQR) (y)	80.0(71.0–83.0)	76.0(70.0–83.0)	0.417	81.5(74.3–84.3)	75.0(70.0–82.0)	0.128	81.0(83.5–74.0)	75.5(69.3–82.0)	0.306
Sex M N(%)	6(50)	64(58)	0.517	10(83)	30(53)	0.047	5(71)	35(56)	0.368
BMI < 18.5 N(%)	5(20)	54(20)	0.734	11(18)	48(21)	0.606	7(57)	52(15)	0.025
Medical history N(%)			0.464			0.082			0.484
Cerebrovascular disease	2(3)	13(19)		4(6)	11(16)		3(4)	12(17)	
Parkinson’s disease	1(1)	10(14)		2(3)	8(12)		1(1)	9(13)	
Dementia	0(0)	7(10)		2(3)	5(7)		0(0)	7(10)	
Gastrointestinal disorders	2(3)	5(7)		1(1)	6(9)		0(0)	7(10)	
Neurological diseases	0(0)	5(7)		1(1)	4(6)		0(0)	5(7)	
Psychiatric disorders	0(0)	4(6)		0(0)	4(6)		0(0)	4(6)	
Pneumonia	0(0)	4(6)		1(1)	3(4)		2(2)	2(3)	
Respiratory diseases	0(0)	3(4)		1(1)	2(3)		1(1)	2(3)	
Spinal canal stenosis	0(0)	3(4)		0(0)	3(4)		0(0)	3(4)	
Others	1(1)	10(14)		0(0)	11(16)		0(0)	11(16)	
FOIS at the time of the first medical examination N(%)			0.463			0.118			0.474
FOIS7	4(6)	38(54)		5(7)	36(52)		4(6)	37(54)	
FOIS6	0(0)	11(16)		3(4)	8(12)		0(0)	11(16)	
FOIS5	1(1)	8(11)		1(1)	8(12)		1(1)	8(11)	
FOIS4	0(0)	6(9)		2(3)	4(6)		2(3)	4(6)	
FOIS2	1(1)	1(1)		1(1)	1(1)		0(0)	2(3)	
Primary complaint at the time of the first medical examination N (%)			0.969			0.763			0.499
Cough while eating	4(6)	31(44)		5(7)	29(42)		5(7)	29(42)	
Weight loss	0(0)	3(4)		1(1)	2(3)		0(0)	3(4)	
Difficulty swallowing	0(0)	13(19)		4(6)	9(13)		0(0)	13(19)	
Catching in the throat (chest)	1(1)	12(17)		0(0)	13(19)		1(1)	12(17)	
Phlegm in the mouth	0(0)	3(4)		1(1)	2(3)		1(1)	2(3)	
Want to eat by mouth	1(1)	2(3)		1(1)	2(3)		0(0)	3(4)	

For the epiglottic vallecula, 17% (*n* = 12) of patients had pill residues. Patients with pill residues had a higher median age of 81.5 (IQR 74.3–84.3) years than those without 75.0(IQR 70.0–82.0) years, though this difference was not statistically significant (*p* = 0.128). Notably, a higher proportion of males was found in the pill residue group (83%) than in the non-residue group (53%), with borderline statistical significance (*p* = 0.047).

For pill residues in the piriform sinus, 10% (*n* = 7) of patients had residues. The median age of these patients was 81.0(IQR 74.0–83.5.0.5) years, compared to 75.5(IQR 69.3–82.0) years in those without pill residues (*p* = 0.306). A significant finding was that 57% of patients with piriform sinus residues had a BMI lower than 18.5 kg/m² compared to only 15% in the no-residue group (*p* = 0.025), indicating that a lower BMI might be associated with a higher risk of pill residues in this location.

Regarding the medical history and FOIS scores at the first examination, no statistically significant differences were found between the groups with and without pill residues at any location. Primary complaints, such as cough when food or saliva went down the wrong way or difficulty swallowing, were similarly distributed between groups, with no significant differences.

### Multivariate analysis

Table 5 presents the results of the multivariate analysis examining factors influencing the presence of pill residues at three anatomical locations: the mouth, epiglottic vallecula, and piriform sinus. In all analyses, females and a BMI ≥ 18.5 kg/m² were used as the reference categories. None of the independent variables, including age, sex, BMI, FOIS score at the first examination, or primary complaint, showed a statistically significant association with the presence of pill residues in the mouth. Age showed an odds ratio of 1.05 (95% CI: 0.94–1.16, *p* = 0.408) and FOIS at the first examination having an odds ratio of 1.28 (95% CI: 0.43–3.86, *p* = 0.657). These findings suggest no strong predictors of pill residue formation in the mouth.

**Table 5:** Multivariate analysis of factors affecting pill residues.

Dependent variable	Independent variable	Partial regression coefficient	*p*	Odds	95%CI	
Mouth	age	0.05	0.408	1.05	0.94	1.16
	sex	0.26	0.813	1.29	0.16	10.78
	BMI < 18.5	0.47	0.720	1.61	0.12	21.50
	FOIS at the first time	0.25	0.657	1.28	0.43	3.86
	Primary complaint	0.27	0.458	1.30	0.65	2.63
Epiglottic vallecula	age	0.05	0.201	1.06	0.97	1.15
	sex	2.70	0.031	14.93	1.23	174.34
	BMI < 18.5	0.33	0.743	1.40	0.19	10.30
	FOIS at the first time	−0.41	0.254	0.66	0.33	1.35
	Primary complaint	0.25	0.390	1.28	0.73	2.26
Piriform sinus	age	0.09	0.124	1.09	0.98	1.22
	sex	1.53	0.202	4.61	0.44	48.15
	BMI < 18.5	2.78	0.016	16.18	1.70	154.30
	FOIS at the first time	−0.16	0.721	0.85	0.34	2.09
	Primary complaint	0.15	0.676	1.17	0.57	2.40

Sex was significantly associated with the presence of pill residues. In the case of the epiglottic vallecula, using females as the reference category, males had significantly higher odds of epiglottic vallecula pill residue (OR for males vs. females = 14.93, 95% CI: 1.23–174.34, *p* = 0.031), indicating that male sex was a predictive factor. Other factors, including age, BMI, FOIS, and primary complaint, did not significantly affect pill residue presence in the epiglottic vallecula, with age having an odds ratio of 1.06 (95% CI: 0.97–1.15, *p* = 0.201).

For pill residues in the piriform sinus, the BMI category was significantly associated with pill residue. Participants with BMI < 18.5 kg/m² had an odds ratio of 16.18 (95% CI: 1.70–154.30, *p* = 0.016) for pill residue compared to those with BMI ≥ 18.5 kg/m², suggesting that low BMI had a predictive association in this cohort. Other variables, such as age, sex, FOIS, and primary complaint, did not show a significant association, with age having an odds ratio of 1.09 (95% CI: 0.98–1.22, *p* = 0.124).

## Discussion

The findings of this study highlight several key aspects of dysphagia management, particularly the challenges patients face when swallowing oral medications. By focusing on simulated pill swallowing using VFSS, we identified significant factors contributing to pill residue in various anatomical regions of the oropharynx and examined how factors such as sex, age, and BMI influence dysphagia outcomes.

Our results revealed that males were more likely than females to experience residual pill residue, particularly in the epiglottic vallecula. Swallowing muscle strength typically weakens with aging in both men and women. However, as men generally have more muscle mass, greater baseline swallowing strength, the higher reserve is lost more abruptly with aging or disease, resulting in more pronounced functional deterioration compared with women^12^. Previous reports suggest men may experience greater age-related decline in specific physiological measures (e.g., swallowing duration and power) compared with women^10,11^. This disparity may be attributed to anatomical and physiological differences that impact swallowing mechanics. For instance, males tend to have larger oropharyngeal structures, potentially affecting the coordination and strength of swallowing muscles, thereby increasing the likelihood of pill residue in specific areas. Multivariate analysis confirmed male sex as a predictor of epiglottic vallecula pill residue, with males showing a higher likelihood of residual pills in this location.

These findings align with previous research, including studies by Logemann et al. and Inamoto et al., which identified sex-related differences in swallowing function and anatomical variations, respectively^5,8^. Additionally, Kaneko et al. reported altered swallowing patterns among males, potentially due to differences in pharyngeal anatomy and muscle dynamics^4^. Our study contributes to this body of evidence by underscoring the need to consider sex-specific factors in dysphagia management strategies.

According to the European Society for Clinical Nutrition and Metabolism consensus, a BMI < 18.5 kg/m² is one stand-alone criterion for diagnosing malnutrition^13^. BMI is commonly used as a marker of undernutrition in healthy populations and in patients with disease^14,15^. BMI also significantly influenced pill residue, particularly in the piriform sinus. Patients with BMI < 18.5 kg/m² had a significantly higher risk of pill residue, highlighting the relationship between malnutrition and impaired swallowing function. Lower BMI is often associated with sarcopenia and muscle weakness, which can impair the coordination and strength needed for effective swallowing. Undernutrition or lower BMI is often associated with poorer swallowing function or recovery. In addition, low BMI can synergize with other factors to worsen swallowing outcomes^9,16^. As a surrogate for overall nutritional reserve, BMI is one of the most commonly used objective nutritional markers for nutritional assessment in dysphagia research^17^.

The association between malnutrition and dysphagia is well-documented. Studies by Garcia Rodríguez et al. and Hägglund et al. found that undernourished patients are more prone to dysphagia-related complications, including difficulty swallowing medications^18,19^. Furthermore, Mañas-Martínez et al. highlighted the importance of adequate nutritional status in preventing the exacerbation of swallowing difficulties in geriatric populations^20^.

Our findings suggest that BMI is a critical factor to consider in the management of dysphagia, especially when administering oral medications. Clinicians should recognize that underweight patients may require tailored interventions, such as modified medication forms or more intensive swallowing rehabilitation, to ensure safe and effective medication administration.

The presence of residual pills in the epiglottic vallecula and piriform sinus, especially among older males and those with lower BMI, has significant clinical implications. Residual pills increase the risk of aspiration, which can lead to aspiration pneumonia–a potentially life-threatening condition, particularly in older adult and frail patients. Therefore, healthcare providers should implement proactive measures to mitigate the risks associated with pill swallowing in patients with dysphagia.

Tailored dysphagia management should consider sex and nutritional status when developing treatment plans. Clinicians may need to pay closer attention to swallowing mechanics in male patients during VFSS studies and consider interventions targeting the epiglottic vallecula and piriform sinus regions. For patients with low BMI, nutritional support and individualized swallowing therapy may be necessary to strengthen swallowing muscles and reduce the likelihood of pill residue.

Additionally, alternative medication forms, such as liquid formulations or crushable pills, may be appropriate for patients at high risk of swallowing difficulties. Combining this approach with dietary modifications and swallowing therapy may significantly improve patient outcomes by reducing the risk of aspiration and promoting medication adherence.

To minimize patient exposure to repeated VFSS swallowing studies, we limited our analysis to a single pill. Therefore, we could not assess whether other pill sizes or forms, such as smaller or coated pills, are easier to swallow. Although this study provides valuable insights into the challenges of pill swallowing in patients with dysphagia, further investigation is warranted. Previous studies exploring the extent to which pill residues occur in patients are limited. This exploratory study is limited by the relatively small sample size and small number of events, increasing the chance of model instability; therefore, our findings should be interpreted with caution. Additionally, studies examining the effectiveness of various interventions, such as modifying pill size, shape, and texture, on reducing pill residue could further guide optimized medication administration for patients with dysphagia.

Further research should also examine the role of cognitive function in pill swallowing, as conditions like dementia and Parkinson’s disease may complicate swallowing instructions and safe ingestion of oral medications. Investigating these factors in larger, more diverse populations would contribute to a comprehensive understanding of the challenges faced by patients with dysphagia and help refine treatment protocols.

## Conclusion

This study identified sex and BMI as significant predictors of pharyngeal pill residue in patients with dysphagia, with males and those with lower BMI being at a higher risk for swallowing difficulties, particularly when taking pills. These findings underscore the importance of comprehensive assessments in dysphagia clinics that incorporate both swallowing and nutritional evaluations to optimize patient care. Given the challenges faced by underweight patients with dysphagia, especially those with BMI < 18.5, non-pill dosage forms should be considered to improve medication adherence and reduce aspiration risk. Safer alternatives, such as sprays, powders, and orally disintegrating pill, may be beneficial. In addition, innovative administration methods such as pill crushing or swallowing aids should be explored to further support safe medication intake in patients with dysphagia.

## Methods

### Patient selection

This retrospective study included patients who visited the outpatient clinic of the Department of Dysphagia Rehabilitation and the Department of Dentistry, Tokyo Medical and Dental University Hospital for dysphagia rehabilitation between May 2013 and March 2020. We included 70 patients who underwent VFSS as part of clinical practice and consented to a swallowing assessment using simulated pills. Patients with severe communication impairments or poor alertness were excluded from the study. In this study, since no prior sample size calculation was performed, post-hoc power was calculated. Under the conditions of a median difference in BMI of 1.1 between the two groups with and without pill residue, a IQR of 0.4, and a significance level of 5%, the calculated post-hoc power was exceeded 0.999.

### VFSS procedure

**Fig. 4:**
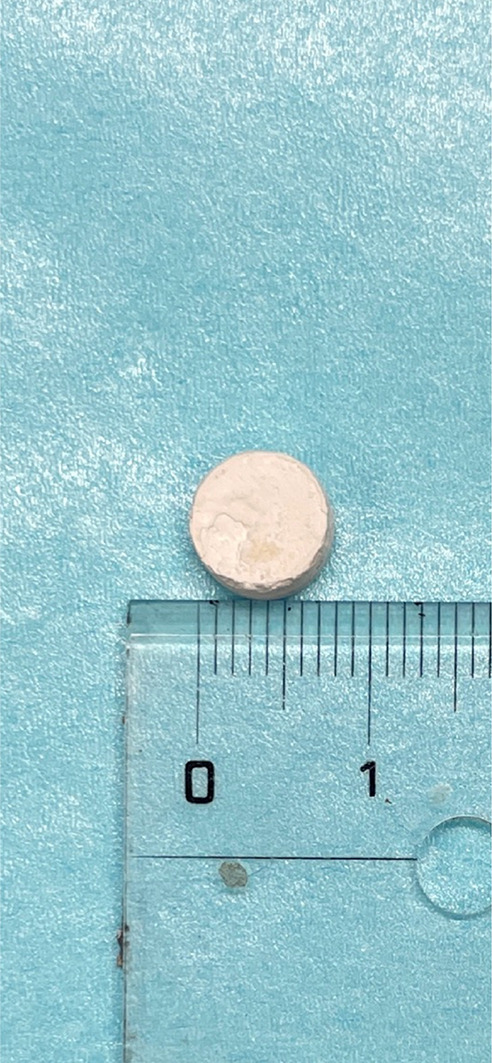
Barium sulfate pill measuring 8 × 4 mm.

Barium mock pill experiments are designed to simulate real-world pill or capsule ingestion and to evaluate the swallowing of solid oral dosage forms under fluoroscopy. Patients were provided with barium placebo pills and a barium solution, and instructed to “take it as you usually do.” Videofluoroscopic examinations were performed using a digital X-ray television system (Shimadzu Medical Systems SDR-100, Osaka, Japan) at a capture rate of 30 frames per second. During the VFSS assessments, simulated barium sulfate pills (Varitop P; Kaigen Pharma, Osaka, Japan) were used. These pills were cylindrical, with a diameter of 8 mm and height of 4 mm (Fig. 4). The pills were placed on each patient’s tongue in a natural sitting position to simulate a typical eating posture. Patients were instructed to swallow the pills along with a barium sulfate suspension, with viscosity adjusted to “drink thickness” consistency as per International Dysphagia Diet Standardisation Initiative classification, using a thickening agent (Tromi Up Perfect, Nisshin Oillio, Tokyo, Japan). Drink thickness for each subject was confirmed in advance as IDDSI levels 0 to 4 to avoid any risk of aspiration. All sites of pill retention after swallowing were evaluated. Within the irradiation fields of the swallowing contrast study, pill residues were observed only in the oral cavity, epiglottic vallecula, and piriform sinus.

### Data collection

Data were collected from VFSS images and medical records, including patient demographics, current medical history, presence or absence of pharyngeal residues of pills, and aspiration of pills and water. Additional variables recorded included the primary complaint at the outpatient visit, BMI, Functional Oral Intake Scale (FOIS) score at the initial visit. Residual pills were assessed at three sites: the oral cavity, epiglottic vallecula, and piriform sinus. A pill was considered residual if it did not pass within 30 s of the swallowing instruction. Aspiration was defined as the entry of food or saliva into the subglottic area. In this study, it was identified as the entry of pills or liquids into the subglottic area. All VFSS data were assessed by a single dentist with 13 years of experience, who has been a certified member of the Japanese Society of Dysphagia Rehabilitation for 5 years.

### Statistical analysis

Statistical analysis was performed using SPSS 28.0 J for Windows (IBM, NY, USA). The Mann-Whitney U and chi-square (X²) tests were used to compare patient demographics, the presence of pill residues, and locations of pill residue. Logistic regression analysis was performed with placebo pills retention in the oral cavity, epiglottic vallecula, and piriform sinus as the dependent variables, and age, sex, BMI < 18.5, FOIS score at the first examination, and primary complaint as independent variables. A p-value of < 0.05 was considered statistically significant.

## Data Availability

The data that support the findings of this study are not openly available due to ethical restrictions. The data are, however, available from the corresponding author upon reasonable request.
