# Correction: Sex and BMI as predictors of pill residue in dysphagia: a multivariate analysis

**DOI:** 10.1038/s41598-026-62551-x

**Published:** 2026-07-27

**Authors:** Ayako Nakane, Mariko Ando, Yu Yoshizumi, Shinya Mikushi, Kazuo Motomura, Haruka Tohara

**Affiliations:** 1 Dentistry and Oral Surgery, Community Healthcare Organization, Tokyo Shinjuku Medical Center, 5-1, Tsukudo-cho, Shinjuku-ku, 162-8543 Tokyo Japan; 2 Department of Dysphagia Rehabilitation, Division of Gerontology and Gerodontology, Graduate School of Medical and Dental Sciences, Institute of Science Tokyo, 1-5-45, Yushima, Bunkyo-ku, 113-8549 Tokyo Japan; 3 Oral Surgery, Saitama Red Cross Hospital, Japanese Red Cross Society, 1-5, Shintoshin, Chuo-ku, 330-8553 Saitama Japan; 4 Morimoto Dental Clinic, 5-11-66 Nameshi, Nagasaki city, 852-8061 Nagasaki Japan; 5 Department of Gerodontology and Oral Rehabilitation, Division of Gerontology and Gerodontology, Graduate School of Medical and Dental Sciences, Institute of Science Tokyo, 1-5-45, Yushima, Bunkyo-ku, 113-8549 Tokyo Japan

Correction to: *Scientific Reports* 10.1038/s41598-026-43307-z, published online 08 March 2026

The original version of this Article contained an error in the order of the Figures:

Figure 4 was typeset incorrectly as Figure 1. The original Figure 1 and accompanying legend appear below:

**Fig. 1:**
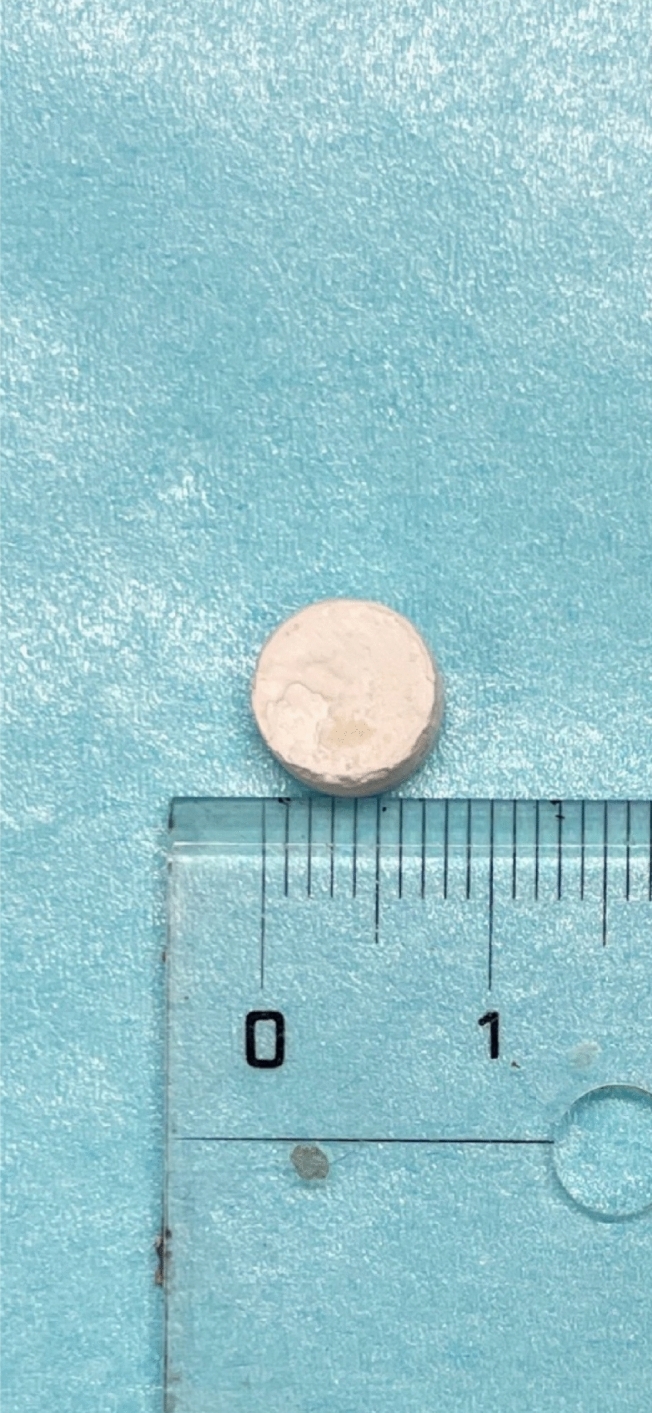
Radiograph, lateral view, showing pill residue in the epiglottis. The arrow indicates the pill residue.

Figure 1 was typeset incorrectly as Figure 2. The original Figure 2 and accompanying legends appear below:

**Fig. 2:**
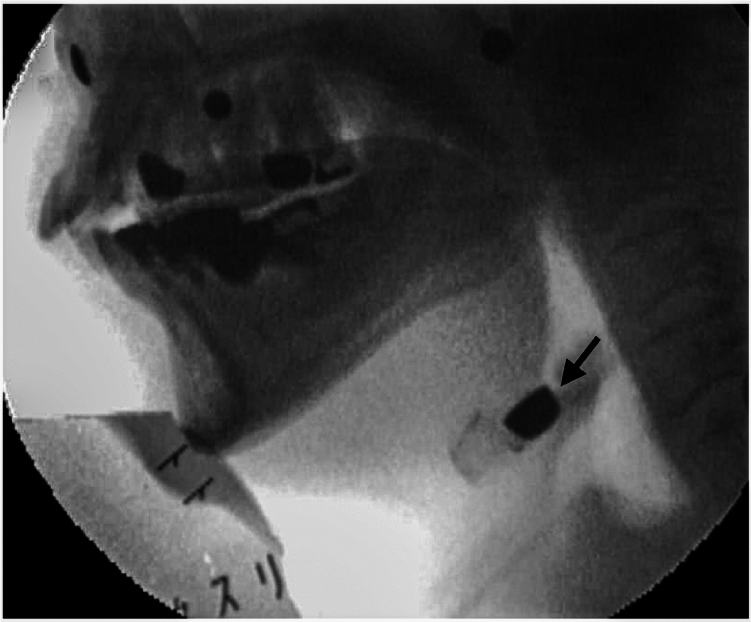
Radiograph, lateral view, showing pill residue in the mouth. The arrow indicates the pill residue.

Figure 2 was typeset incorrectly as Figure 3. The original Figure 3 and accompanying legends appear below:

**Fig. 3:**
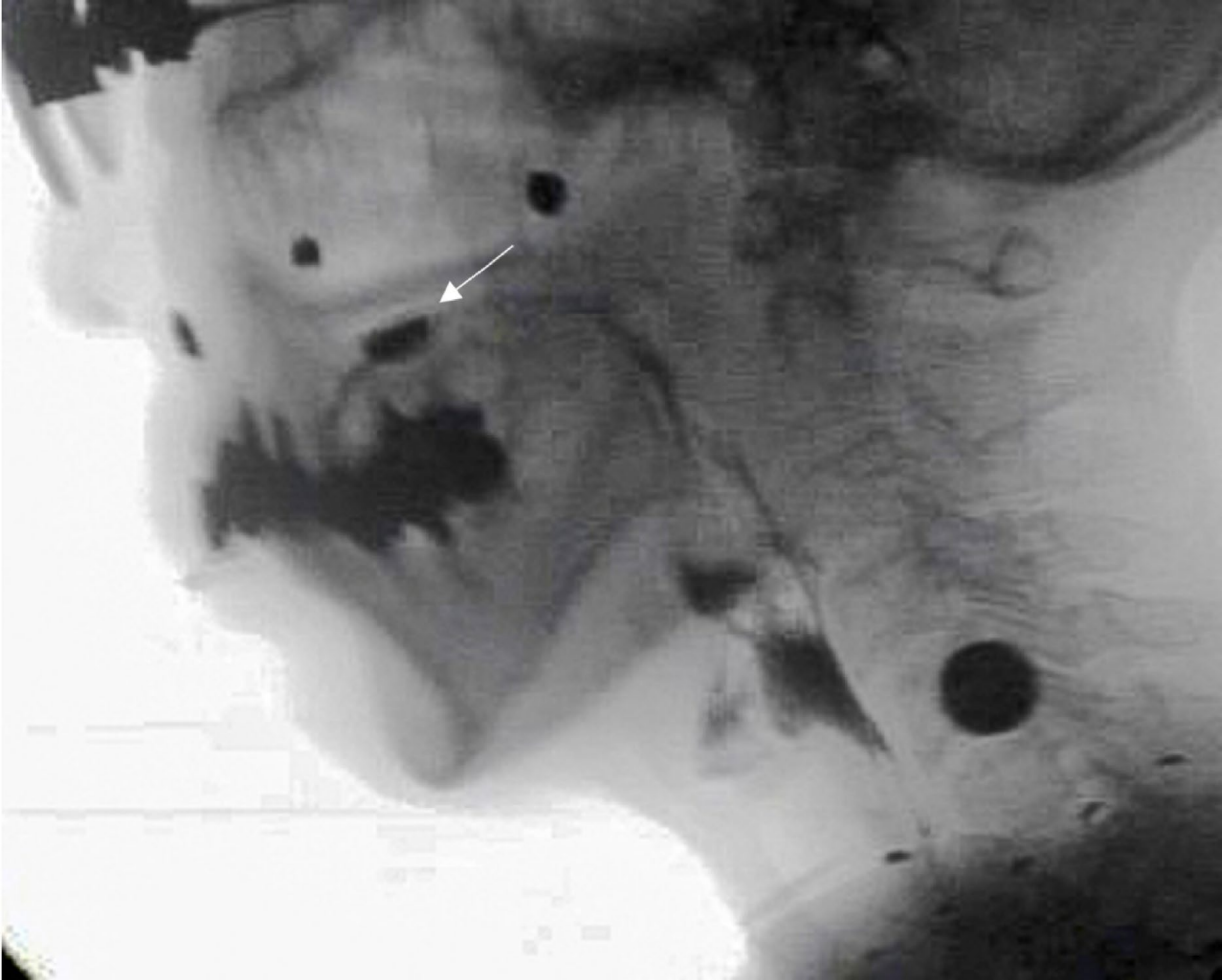
Radiograph, lateral view, showing pill residue in the piriform sinus. The arrow indicates the pill residue.

Figure 3 was typeset incorrectly as Figure 4. The original Figure 4 and accompanying legends appear below:

**Fig. 4:**
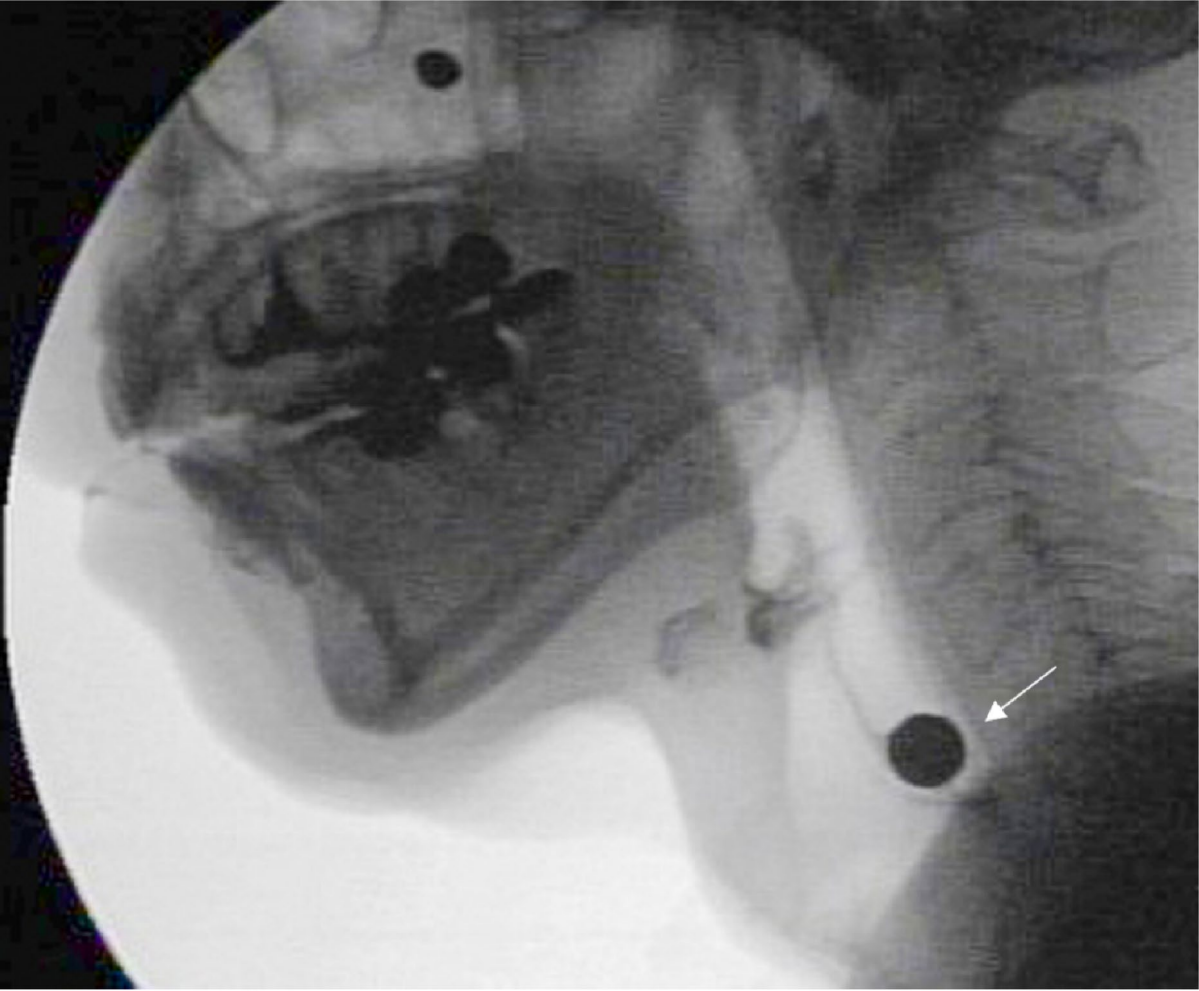
Barium sulfate pill measuring 8 × 4 mm.

The original Article has been corrected.

